# The potential of plant extracts in cell therapy

**DOI:** 10.1186/s13287-022-03152-z

**Published:** 2022-09-14

**Authors:** Caifeng Li, Zhao Cui, Shiwen Deng, Peng Chen, Xianyu Li, Hongjun Yang

**Affiliations:** 1 Beijing Key Laboratory of Traditional Chinese Medicine Basic Research On Prevention and Treatment for Major Diseases, Experimental Research Center, China Academy of Chinese Medical Sciences, Beijing, 100700 China; 2 Institute of Chinese Materia Medica, China Academy of Chinese Medical Sciences, Beijing, 100700 China; 3 Robot Intelligent Laboratory of Traditional Chinese Medicine, Experimental Research Center, China Academy of Chinese Medical Sciences and MEGAROBO, Beijing, China

**Keywords:** Cell therapy, Plant extracts, Stem cell, CAR-T, TCR-T

## Abstract

Cell therapy is the frontier technology of biotechnology innovation and the most promising method for the treatment of refractory diseases such as tumours. However, cell therapy has disadvantages, such as toxicity and poor therapeutic effects. Plant extracts are natural, widely available, and contain active small molecule ingredients that are widely used in the treatment of various diseases. By studying the effect of plant extracts on cell therapy, active plant extracts that have positive significance in cell therapy can be discovered, and certain contributions to solving the current problems of attenuation and adjuvant therapy in cell therapy can be made. Therefore, this article reviews the currently reported effects of plant extracts in stem cell therapy and immune cell therapy, especially the effects of plant extracts on the proliferation and differentiation of mesenchymal stem cells and nerve stem cells and the potential role of plant extracts in chimeric antigen receptor T-cell immunotherapy (CAR-T) and T-cell receptor modified T-cell immunotherapy (TCR-T), in the hope of encouraging further research and clinical application of plant extracts in cell therapy.

**Supplementary Information:**

The online version contains supplementary material available at 10.1186/s13287-022-03152-z.

## Introduction

As the frontline of biotechnological innovation, cell therapy has a significant influence on medical treatment and provides new sights for difficult diseases [1]. Cell therapy mainly includes stem cell therapy and immune cell therapy. Stem cells can be acquired from embryonic tissue, foetal tissue, adult organism, and induced pluripotent stem cells (iPSCs) [2, 3]. According to developmental stage, stem cells include pluripotent stem cells (PSCs) and adult stem cells. Among them, PSCs include embryonic stem cells (ESCs) and iPSCs [3], which are pluripotent. However, ESCs have ethical issues, and the probability of reproductive cloning and tumour formation limited the application of iPSCs [3]. Adult stem cells overcome these problems of PSCs, which contain mesenchymal stem cells (MSCs), neural stem cells (NSCs), and adipose-derived stem cells (ASCs), among others, and have significant therapeutic effects in cardiovascular, neurological, skeletal, and autoimmune diseases [4, 5]. In addition, immune cell therapy, represented by chimeric antigen receptor T-cell immunotherapy (CAR-T), and T-cell receptor modified T-cell immunotherapy (TCR-T), has shown potential efficacy in multiple myeloma and other haematologic malignancies as well as in some solid tumours [6]. Cell therapies have a large latent capacity under the development of modern science and technology. However, cell therapy itself still has deficiencies in practice, such as side effects, inflammatory factor storms caused by excessive immune responses and poor effects in some patients. As a result, combining it with other drugs may be a way to solve this problem. Additionally, protein cytokines and antibodies have been widely used in cell culture and clinical treatment, with disadvantages such as toxicity, and high cost. Therefore, at present, looking for alternative plant extracts that can be used as growth factors and adjuvant therapies in cell therapy is a promising approach [7].

Hormesis effects on stem cells have been observed, mainly in the use of drugs (e.g. metformin, atorvastatin, and isoproterenol), dietary supplements/medicinal plant extracts (e.g. berberine), and endogenous drugs (e.g. estrogen) [8, 9]. Among them, plant extracts are an important source of bioactive small molecules, which are mainly derived from herbal plants, and include flavonoids, alkaloids, polysaccharides, volatile oils, etc. Plant extracts play an important role in disease treatment, especially in cancer and infectious diseases. Herbal therapy is a traditional medical practice that has long been used to treat a variety of diseases. Herbal medicine is safe and affordable [7]. It is a promising alternative approach with a significant effect on alleviating patient disease. Therefore, studying the effects of plant extracts in cell therapy and digging out active plant extracts in herbal medicines can provide guidance for adjuvant therapy combined with cell therapy.

The application of plant extracts in cell therapy has gradually increased with the rapid development of cell therapy. For example, the increased therapeutic effects of stem cells induced by plant extracts have been reported in studies of Alzheimer’s disease [10], chronic kidney disease [11], and stroke [12], and the therapeutic effects of immune cells induced by plant extracts have been reported in diseases such as non-small-cell lung cancer and liver cancer [13, 14]. This review describes the effects of plant extracts, mainly herbal extracts, in cell therapy approaches and clarifies the potential mechanisms of action when possible.

## The effect of plant extracts on stem cell therapy

### The effect of plant extracts on MSCs

**Fig. 1:**
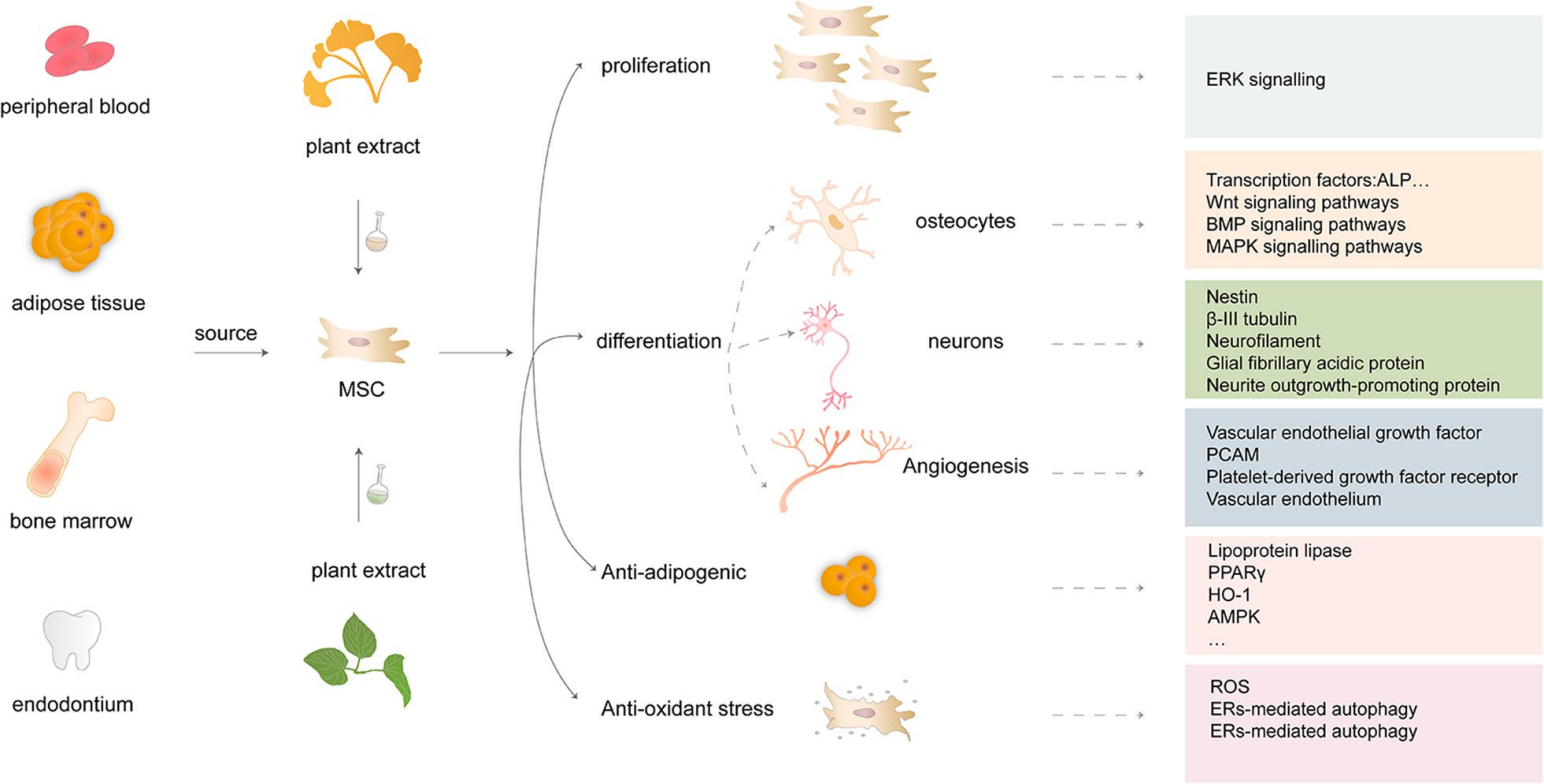
Sources of MSCs and their proliferation, differentiation, angiogenesis, antilipogenesis, and antioxidant stress effects stimulated by plant extracts

MSCs exist in almost all postnatal human tissues. The major sources of adult MSCs are mainly from bone marrow, adipose tissue, etc. [7, 15]. Compared with ESCs, MSCs are easier to isolate and culture in vitro, and more importantly, there are fewer ethical issues. Furthermore, due to their HLA-DR-negative feature, MSCs do not have immunogenic in therapy [7]. MSCs are characterized by different sources, isolation methods, and epigenetic changes during growth. They can be differentiated into osteocytes, neurons, and angiogenesis, through stimulation with plant extracts (Fig. 1).

### Proliferation effect

Many plant extracts, such as *Foeniculum vulgare* [16]*, Ferula gummosa* [17], amentoflavone (*Selaginella tamariscina* (P. Beauv.) Spring) [18], gastrodin (*Gastrodia elata* Bl.) [19], and resveratrol (*Polygonum cuspidatum* Sieb. et Zucc.) [20], can significantly increase bone marrow-derived human MSCs (BM-hMSCs) proliferation. In addition, ginsenoside Rg1, which is an effective compound in *Panax ginseng*, *Panax notoginseng*, and American ginseng, can also promote cell proliferation [21]. Apple ethanol extract promotes proliferation of hASCs and human cord blood-derived MSCs via ERK signalling [22]. *Tinospora cordifolia* and *Withania somnifera* are traditional Ayurveda medicinal materials in India that are reported to improve cell proliferation ability and activity, as well as reduce cell apoptosis and postpone aging [23]. ZD-I is a prescription composed of seven traditional Chinese medicines (TCM). It has stimulatory effects on the proliferation of hMSCs [24]. *Viscum album* induces primitive placenta-derived MSCs (PDSCs) with remarkable proliferative properties through autophagy mechanism. Specifically, *Viscum album* can regulate the cell cycle to make PDSCs self-renewal and regulate the induction of survival factors, apoptosis and autophagy to reduce cell death [25].

## Differentiation effect

### Osteogenic effects

#### Osteogenic effects via transcription factors

*Foeniculum vulgare, Ferula gummosa,* and amentoflavone can significantly increase the alkaline phosphatase (ALP) activity of hMSCs and promote BM-hMSC differentiation into osteoblasts [16–18]. *Dipsacus asper* and its ingredients hedraganin-3-*O*-(2-*O*-acetyl)-α-l-arabinopyranoside enhance osteoblastic differentiation not only by inducing ALP activity but also by inducing bone sialoprotein and osteocalcin expression [26]. Moreover, *Fructus Ligustri Lucidi* effectively activated ALP, reduced the osteogenic differentiation time of MSCs, and up-regulated the expression of osteogenic related factors such as catenin, BMP2, cyclin D1, membrane matrix metalloproteinase, osteoprotegerin and T-box 3 [27]. Poncirin (*Poncirus trifoliata* (L.) Raf) [28], *Panax notoginseng* saponins [29, 30], and naringin (*Citrus grandis*) [31, 32] decreased peroxisome proliferator-activated receptor γ (PPARγ) 2 mRNA levels, while *Panax notoginseng* saponins raised the levels of ALP, Cbfa 1, OC, BSP, OPG, β-catenin, and cyclin D1. Harmine (*Peganum harmala* L.) increased ALP activity and up-regulated osteocalcin expression. Moreover, harmine can up-regulate osterix [33]. The combination of epigallocatechin-3-gallate [34] and bone inducer can up-regulate BMP2 and enhance bone formation. Gastrodin [19] improved ALP, OCN, COL I, and OPN while reducing ROS. Fucoidan enhanced osteogenic specific marker genes, such as ALP, osteopontin, type I collagen, Runx2, and osteocalcin in ASCs [35]. Quercetin (*Sophora flavescens* Ait.) can increase Osx, Runx2, BMP2, Col1, OPN and OCN, and enhance osteogenic differentiation [36]. BuShenNingXin decoction (BSNXD) up-regulated ALP and collagen type I, osteocalcin, Runx2, and osterix. Furthermore, BSNXD was shown to reduce the quantity of adipocyte and PPARγ mRNA [37]. A summary table of plant extracts that stimulate osteogenesis of MSCs is shown in Additional file 1: Table S1.

### Osteogenic effects through Wnt signalling pathways

**Fig. 2:**
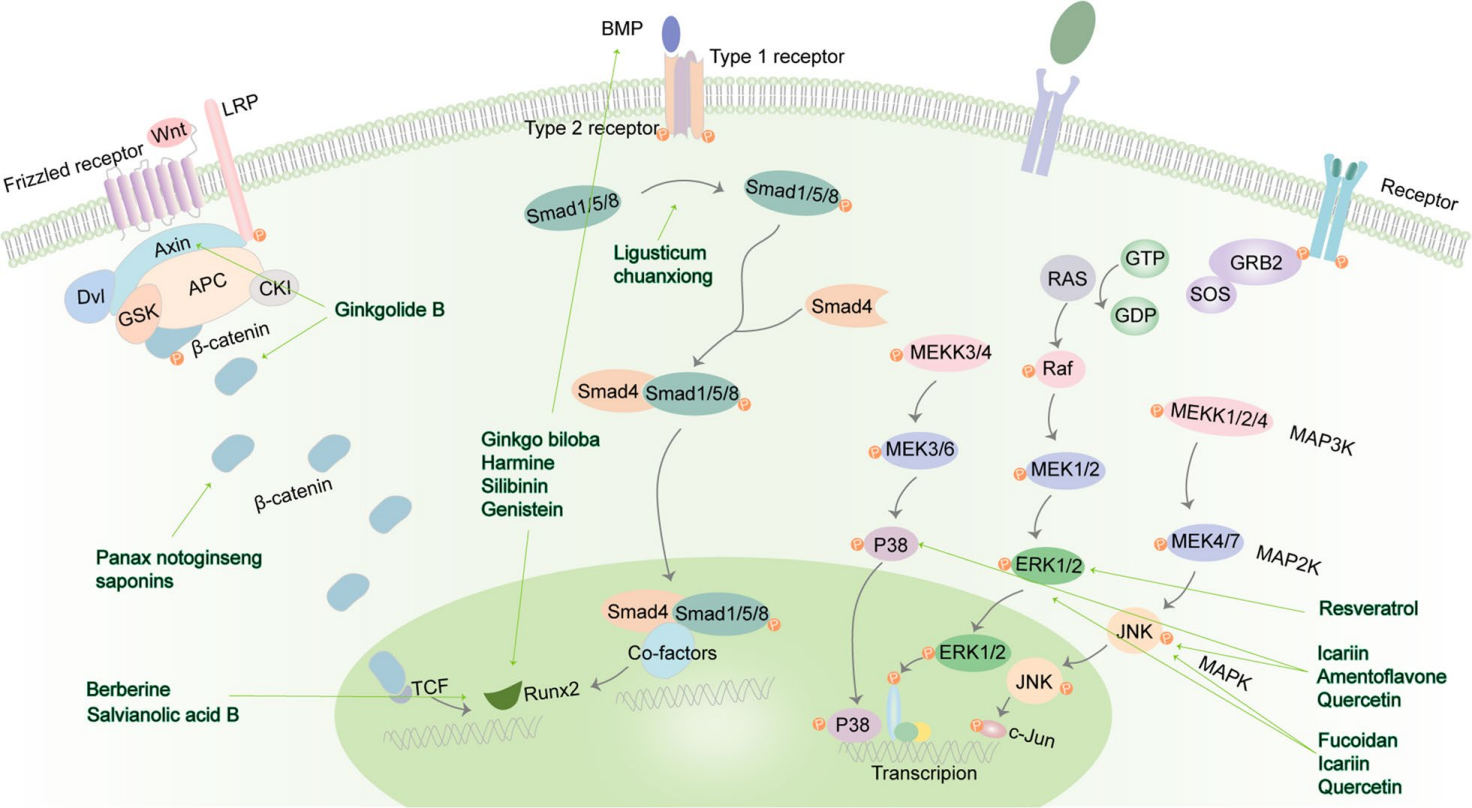
Plant extracts that affect MSCs osteogenesis by regulating intracellular signalling pathways. Ginkgolide B, *Panax notoginseng* saponins, berberine, and salvianolic acid B regulate axin, β-catenin, and TCF in the Wnt signalling pathway; *Ginkgo biloba*, harmine, silibinin, genistein, and *Ligusticum chuanxiong* regulate BMP, Runx2 and Smad 1/5/8 in the BMP signalling pathway; Resveratrol, icariin, amentoflavone, quercetin, and fucoidan regulate p38, ERK1/2, and JNK in the MAPK signalling pathway

Flavonoids of epimedii(*Epimedium brevicornum* Maxim., etc.) were found to increase the rates of osteogenic activity through the BMP or Wnt-signalling pathway [38]. In addition, *Angelica sinensis* polysaccharide can enhance the osteogenic differentiation of rat BM-MSCs cultured in high-sugar and guide bone regeneration in type 2 diabetes animal model which chained to the Wnt/β-catenin signalling pathway [39]. *Ginkgo biloba* and its main component ginkgolide B accelerate osteoblast differentiation and the formation of bone via Wnt/β-Catenin signalling [40] (Fig. 2). Berberine (*Coptis chinensis* Franch.) [41] and salvianolic acid B (*Salvia miltiorrhiza* Bge.) [42, 43] promote osteogenesis in BM-MSCs through Wnt/β-catenin signalling and strengthen Runx2 expression. Salvianolic acid B influences the ERK signalling pathway and lower PPARγ mRNA, accelerating the osteogenesis of MSCs. The osteogenic-related genes can be strengthened under the induction of naringin, and the expression of Notch1 can be up-regulated at the same time, and activation Wnt signalling activation [44].

### Osteogenic effects through BMP signalling pathways

*Ginkgo biloba* has been found to enhanced Runx2 expression and regulated BMP4 in BMP signalling [45]. Moreover, harmine [33], silibinin(*Silybum marianum* (L.) Gaertn.) [46], and genistein(*Genista tinctoria* Linn., etc.) [47] activate the BMP and Runx2 pathways. Duhuo Jisheng decoction and its effective component *Ligusticum Chuanxiong* can activate Smad 1/5/8 and ERK signalling, increase the osteogenic effect of MSCs, and improve BMP-2 and Runx2 [48] (Fig. 2).

### Osteogenic effects through MAPK signalling pathways

Most of the plant extracts used in the study of MSCs osteogenesis are TCM monomers. Icariin (*Epimedium brevicornum* Maxim., etc.), amentoflavone and quercetin promoted osteogenesis via the JNK and p38 MAPK pathways (Fig. 2). Icariin also phosphorylates ERK, and stimulates PI3K-AKT-eNOS-NO-cGMP-PKG pathway in bone marrow stromal cells [18, 49, 50]. Quercetin is a flavonoid that can also activate ERK signalling pathways, decrease the aging and oxidative stress in MSCs, and promote osteogenic differentiation [51, 52]. Fucoidan can induce osteogenic differentiation, activate ERK and JNK mainly through BMP2 Smad 1/5/8 signalling, and regulate osteogenic differentiation markers [53, 54]. One study showed that resveratrol enhanced cell renewing by inhibiting cell aging at a low concentration, while it inhibits cell self-renewal by up-regulating cell senescence, doubling time, and S-phase arrest at a high concentration. In addition, it can stimulate MSCs and promote osteoblast differentiation by acting on ER-dependent mechanisms and activating ERK1/2 [20, 55, 56].

### Neurogenic effects

*Mucuna gigantea* grows natively in Hawai ‘i. It was recorded that it can be used to treat kampavata (excitatory paralysis) [57]. Mucuna gigantea can promote proliferation feature, nestin, and β-III tubulin mRNA expression in MSCs [58].

A study using human umbilical cord Wharton's Jelly-derived MSCs (WS-MSCs) showed that *Salvia miltiorrhiza* increases the expression of nestin, β-tubulin, neurofilament, GFAP, and neurite outgrowth-promoting protein [59]. Another study in rat BM-MSCs showed that *Salvia miltiorrhiza* promotes Mash-1 and NGN-1 induced mRNA expression of TUJ-1, NF, and synaptophysin [60].

Ginkgolide B and *Astragalus mongholicus* can increase NSE-positive neuron-like cells and GFAP-positive astrocyte-like cells to promote MSCs differentiation into nerve cells [61, 62]. Moreover, *Astragalus mongholicus* also enhances the expression of the Wnt-1 gene and Ngn-1 gene [62]. Ginsenoside Rg1 was found to accelerate the differentiation of neural phenotype in hASCs and upregulate NSE, MAP-2, GAP-43, NCAM, and SYN-1 genes [21]. Another study showed that gisenoside Rg1 can promote neural differentiation in mouse ASCs by miRNA-124 signalling pathway [63]. Similarly, radix *Angelicae sinensis* can also induce adipose-derived MSCs to differentiate into neuron-like cells [64]. In Ayurvedic medicine, dhanwantharam kashayam is considered a growth stimulant for children, which can promote nerve regeneration [65].

### Angiogenesis effects

Treatment of hMSCs with olive leaf extract promoted the differentiation of cells into endothelial cells and development of the tubular construction needed for angiogenesis. At the same time, olive leaf extract can promote VEGF, PCAM, PDGF, and VEGFR-1 [7]. Curcumin is an antioxidant and anti-inflammatory substance in turmeric. Its ethanol extracts can increase the expression of CD34, CD133, and VEGFR2 to cause ASCs to proliferation and differentiation into endothelial progenitor cells [66]. In the animal hindlimb ischaemia model, fucoidan can protect MSCs from oxidative stress and enhance angiogenesis. Another study showed that fucoidan can inhibit the cell death caused by MSCs ischemia, and adjust the levels of apoptosis-related proteins and cellular ROS mainly by MnSOD and Akt pathways [67]. In addition, through ERK-IDO-1 signalling cascade, it increases the proliferation potential and the expression of cell cycle-associated proteins, and enhances the immunoregulation activity of MSCs [11]. Carica papaya leaf extract, rich in papain, was found to enhance the composition of IL-6 and stem cell factors related to platelet production in vitro [68].

### Anti-adipogenic effects

Some plant extracts also have anti-adipogenic effects on MSCs. The results of one study confirmed that the antioxidant action of *Tithonia diversifolia* may influence the expression of HO-1. More importantly, it may regulate carbohydrate and fat metabolism by repressing adipocyte differentiation through activating AMPK [69]. In an experiment using MSCs, after stimulation with aloe-emodin, many indicators were reduced, including resistin, adiponectin, aP(2), lipoprotein lipase, PPARγ, and TNFα, which influence adipogenic pathways [70]. Quzhisu can repress adipogenic differentiation of BM-MSCs by downregulating PPARγ [71]. Similarly, flavonoids of epimedii like Quzhisu downregulate PPARγ, and can also decrease C/EBP-α [72].

### Antioxidant stress effects

*Undaria pinnatifida*, *Myrtus community* L. and *Cirsium setidens* showed antioxidant stress effects in MSCs. *Undaria pinnatifida*, also called Mi-Yoek in Korea, is considered a healthy food. The anti-aging effect of *Undaria pinnatifida* in BM-MSCs was researched. The results showed that after H_2_O_2_ treatment, it had the effect of antioxidant stress, and could decrease aging and improve the differentiation potential of cells by controlling ROS [73]. In addition, icariin protected rabbit BM-MSCs from oxygen, glucose, and apoptosis via inhibition of ERs-mediated autophagy associated with MAPK signalling [74]. Furthermore, residues from the production of *Myrtus community* L. can counteract the appearance of aging phenotypes in ASCs, reduce oxidative stress and inflammation, and enhance the expression of genes related to pluripotency [75]. The authors also studied the genetic programs responsible for cellular senescence in human ASCs exposed to oxidative stress and found that in the cells stimulated by Myrtle, the SA-β-Gal positive cells and the cell cycle regulation genes were decreased, while TERT and c-Myc genes were increased [76]. *Cirsium setidens* [77] has a suppressive effect on cell injury by regulating oxidative stress and repressing apoptosis-related signalling pathways.

### Adverse

The above studies have shown that certain plant extracts can promote the proliferation and differentiation of MSCs. However, some studies have demonstrated that plant extracts have side effects. *Cimicifugae Rhizoma*, also called Shengma in China, affects the vita of dental stem cells, and has side effects on the oral cavity at a high content [78]. Additionally, *Asiasarum* radix is the same as *Cimicifugae Rhizoma* [79]. As mentioned above, *Fructus Ligustri Lucidi* has an osteogenesis effect. Nevertheless, *Fructus Ligustri Lucidi* in a dose of more than 200 μg/mL has cytotoxicity to MSCs [27].

### The effect of plant extracts on NSCs

Endogenous NSCs are abundantly in the subventricular region of the hippocampal granular area and the germinal area of the cerebrum. They differentiate cells according to the needs of brain structure and function [80]. NSCs are primarily used to remedy central nervous system injury and degenerative diseases. There are two intervention strategies involving NSCs. One strategy involves using endogenous NSCs to repair the diseased site, but endogenous NSCs are not sufficient and prefer to differentiate into gliocytes instead of neurons; the other strategy is transplantation of exogenous NSCs [61]. However, it is difficult to control the survival, replication, and differentiation of exogenous NSCs into local nerve cells. Sources of NSCs include human ESCs, human iPSCs, human foetal brain-derived neural stem/progenitor cells, and direct reprogramming of astrocytes. According to their functions, they can be divided into pluripotent and multipotent cell types [81]. Plant extracts play an important part in promoting the proliferation and differentiation of NSCs into new neurons. The Notch, Wnt, BMP, and sonic hedgehog signalling pathways have been the most studied [82].

### Proliferation effect

Ginsenosides Rg1 advances the incorporation of Bromo-2-deoxyuridine and the expression of nestin and vimentin in NSCs, and promotes the proliferation of NSCs [83]. In addition, ginsenoside Rd can enhance the proliferation of NSCs in vivo and in vitro. It can enhance the size and quantity of neurospheres [84]. After oxygen and glucose deprivation (OGD) /r injury in vitro, resveratrol up-regulated the survival and proliferation of NSCs, and increased patched-1, smoothened (SMO) and Gli-1 [85]. Meanwhile, resveratrol can reduce the damage and raise the proliferation of NSCs by promoting Nrf2, HO-1 and NQO1 [86]. Artesunate is a derivative of artemisinin from Artemisia annua [87]. It can inhibit transcription by inducing Foxo-3a phosphorylation, then downregulating p27kip1, and enhancing the proliferation of NSCs in the infarcted cortex through PI3K/AKT signalling transduction [88].

### Differentiation effect

#### Wnt/β-catenin pathway

The role of ginkgolide B has been mentioned above, it also can enhance the differentiation of NSCs after cerebral ischemia and may improve neural function by increasing the expression of BDNF, EGF, and SOCS2 [12]. *Ginkgo biloba* extract and Ginkgolide B, was found to accelerate cell cycle exit and neuronal differentiation in NSCs. Furthermore, ginkgolide B up-regulated the nuclear level of β-catenin and activated the classical Wnt to promote neuronal differentiation [89]. Curcumin (*Curcuma aromatica* Salisb.) has some problems with pharmacokinetics and pharmacodynamics. Thereby Tiwari SK et al. prepared curcumin nanoparticles and found that they can activate the classic Wnt/β-catenin pathway to lead to human neurogenesis [90]. Icariin is an important biologically active ingredient extracted from Epimedium and has neuroprotective properties. Icariin treatment enhanced NSCs neurosphere formation and promoted the expression of nestin, β-III-tubulin and GFAP. Icariin-regulated genes participate in pathways including the Wnt and bFGF signalling [91], ERK/MAPK signalling [92], and BDNF-TrkB-ERK/Akt signalling pathway [93].

### PI3K/AKT signalling pathway

One study evaluated the function of salvianolic acid B on the differentiation, proliferation, and neurite growth of mouse NSCs. The proper dose of salvianolic acid B promoted the quality of NSCs and neurospheres, and accelerates the growth of neurites of NSCs and their differentiation into neurons [94]. Zhuang P et al. selected 45 kinds of ingredients from TCM widely applied in the clinical treatment of stroke in China and examined their proliferation-inducing activity on NSCs. Finally, it was found that salvianolic acid B maintains NSCs self-renewal and promotes proliferation through the PI3K/Akt signalling pathway [95]. Salidroside is an ingredient extracted from the plant *Rhodiola rosea* L. It can inhibit hypoxic NSCs injury by increasing miR-210, thereby repressing BTG3 and influencing PI3K/AKT/mTOR signalling pathway [96]. The protective effect of berberine on OGD-treated cells via inhibiting the cell cycle. It can decrease cyclin D1, p53 and caspase 3, increase the phosphorylation level of p-Bad/tBad, and upregulate PI3K and Akt [97].

### BMP signalling pathway

( +)-Cholesten-3-one(*Serratula*) induced NSCs differentiation into dopaminergic neurons and promoted tyrosine hydroxylase, dopamine transporter, dopa decarboxylase, dopamine secretion, and evidently increased BMPR IB. The p-Smad1/5/8 expression indicates that ( +)-Cholesten-3-one may influence the BMP signalling [98].

### Notch signalling pathway

Astragaloside IV is an ingredient in *Astragalus membranaceus*. Astragaloside IV leads NSCs to β-tubulin III ( +) and GFAP ( +) cells through the Notch signalling pathway [10]. Moreover, in an in vivo study, astragaloside IV can promote proliferative cells(BrdU^+^), premature neurons (DCX^+^), early proliferative cells (BrdU^+^/DCX^+^), proliferative radial Glia-like cells (BrdU^+^/GFAP^+^), and regulate the homeostasis of the CXCL1/CXCR2 signalling pathway [99].

### Others

*Panax notoginseng* saponins notably increased NSCs proliferation and the expression of nestin/BrdU, Tuj-1, and vimentin mRNA in hippocampal NSCs. And the results indicate that *Panax notoginseng* saponins may promote the proliferation and differentiation of NCSs after OGD in vitro by increasing the area density, optical density and the number of nestin/BrdU, nestin/vimentin, and nestin/tuj-1 positive cells [100]. One study investigated the effects of tetramethylpyrazine, an active element of *Ligusticum Chuanxiong*, which promotes the differentiation of NSCs into neurons, increases the phosphorylation of ERK1/2, and reduces the phosphorylation of p38 [101]. Baicalin could increase MAP-2 positive cells and decrease the number of GFAP stained cells. Meanwhile, p-STAT3 and Hes1 were downregulated, and NeuroD1 and Mash1 were upregulated. These results suggested that baicalin can promote neural differentiation but inhibit the formation of glial cells. Its role in promoting neurogenesis is related to STAT3 and bHLH genes [102, 103]. Earlier research on NSCs showed that Buyanghuanwu decoction can promote cell growth and differentiation, increase neurofilament (NF) positive cells and GFAP positive cells, and promote intracellular Ca^2+^ concentrations [104, 105]. Jiaweisinisan has antidepressant effects, promotes hippocampal neurogenesis after stress damage, and significantly increases nestin, β-tubulin-III, and GFAP [106].

### The effect of plant extracts on ESCs

ESCs can be obtained from the inner cell mass of a blastocyst. It has the characteristics of in vitro culture capacity, immortal cell proliferation, self-renewal, and multidirectional differentiation [107]. Using ESCs to differentiate into different cell models is a promising drug discovery method and technology [108]. Kami-Shoyo-San is a TCM that can protect neuronal apoptosis in ESCs by promoting brain-derived neurotrophic factor/tropomyosin receptor kinase B signalling pathway [109].

### The effect of plant extracts on iPSCs

iPSCs have characteristics similar to those of ESCs in terms of unlimited self-renewal and differentiation capabilities. Plant extracts induce iPSCs production and apoptosis. The Sagunja-tang herbal formula can efficiently produce iPSCs from human foreskin fibroblasts via transcription factors [110]. *Prunellae Spica* and *Magnoliae cortex*-mediated apoptosis of undifferentiated iPSCs was found to be p53-dependent, and to have potent anti-teratoma activity, with no genotoxicity toward differentiated cells. Therefore, these compounds can be used for iPSC-based cell therapy to induce apoptosis of possible undifferentiated iPSCs and prevent the occurrence of teratomas [111, 112].

Plant extracts can induce differentiation of iPSCs into nerve cells. *Salvia miltiorrhiza* can significantly increase the expression of nestin and microtubule-associated protein 2 (MAP2) genes and proteins, and induce the differentiation of iPSCs into neurons [113]. Plant extracts also have an improved effect on the nerve cell model differentiated from iPSCs. N-Butylidenephthalide (*n*-BP) is derived from *Angelica Sinensis*. *N*-BP can reduce Aβ40 deposits, total tau protein, and its hyperphosphorylated form in iPSCs-derived neurons induced by Down syndrome [114]. Graptopetalum paraguayense can improve AD-related phenotypes, such as reducing Aβ 40, Aβ 42, and tau protein phosphorylation [115].

iPSCs are differentiated into cardiomyocytes, which are used in the research of related diseases. One study found that *Salvia miltiorrhiza* and *Crataegus pentagyna* have anti-arrhythmic effects. *Salvia miltiorrhiza* has an antioxidant effect, regulates calcium treatment on myocardial cells during I/R and decreases arrhythmia and apoptosis [116]. *Crataegus pentagyna* extract has an anti-arrhythmic effect on cardiomyocytes derived from human arrhythmia-specific iPSCs [117]. In addition, Yixinshu capsule has a protective effect on human iPSCs-derived cardiomyocytes by reducing endothelin 1 (ET-1) induced contractile dysfunction, increasing brain natriuretic peptide (BNP) content, and inducing morphological changes [118]. However, some plant extracts are toxic to cardiomyocytes, such as liensinine and neferine [119], mitragynine [120], and Erythrina senegalensis DC [121].

### The effect of plant extracts on other stem cells

Ultraviolet-B (UVB) irradiation can damage the epidermis. *Andrographis paniculata* [122] promotes the proliferation of epidermal stem cells (EpSCs) and anti-aging via increasing integrin β1 and VEGF expression. Morin [123] and Vanillin [124] significantly inhibited UVB-induced damage to human keratinocyte stem cells, and effectively enriched the p53- specific ligasing ability of the mouse double minute 2 homologue in UVB irradiation-induced p53 activation. Likewise, zingerone (*Zingiber officinale* Rosc.) [125] can protect the epidermis by restraining the UV damage mediated by p42/44 MAPK and p38 MAPK. In other stem cells, *Ginkgo biloba*[126, 127] activates telomerase through PI3k/Akt signalling pathway to delay the aging of endothelial progenitor cells. Additionally, starting from telomerase, TSY-1 [128] increases telomerase activity in CD34^+^ haematopoietic stem cells.

### Immune cell therapies

Adoptive cell therapy (ACT) is a kind of immunotherapy that is genetically modified T-cells to deliver a CAR or TCR. To a certain extent, mutated cancer cells provide many peptides that are not found in natural cells, which brings a potential target for constructing a new antigen screening system, and promotes the development of ACT, making CAR-T and TCR-T treatment become the most prospective way to treat cancer. However, ACT has great differences in the treatment of various tumours types, and there are still some shortcomings that need improvement [129].

### CAR-T

**Fig. 3:**
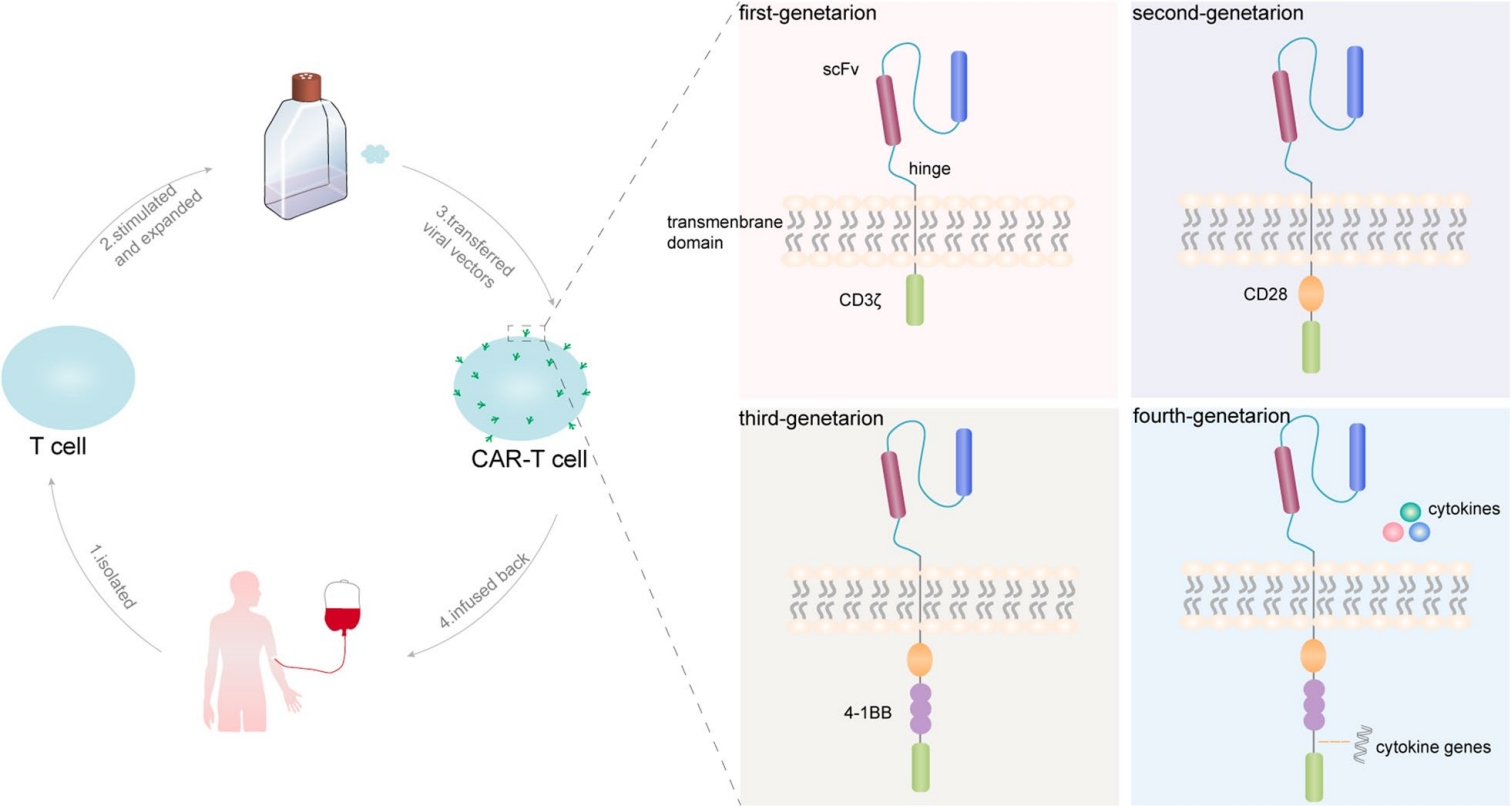
CAR-T-cell therapy and the four generations of improvements. The first-generation CARs were fused with a single-chain variable fragment (scFv) to a transmembrane domain and an intracellular signalling unit: the CD3 zeta chain. Then, the second-generation CARs improved the costimulatory molecule receptor-like CD28, which is the most commonly used. The second-generation CARs increased the production of cytokines and enhanced durability. The third-generation of CARs design in-corporated an additional costimulatory domain to enhance CAR function and included the scFv, the initial CD3ζ-chain, and the CD28 and 4-1BB or OX40 costimulatory domains [132]. At present, fourth-generation CAR-T therapy has been extended. In this type of CAR-T-cell therapy, cytokine genes have been added to the structure, which can stimulate high cytokines expression that enhances the activity of T-cells after CAR-T-cells are activated, thereby improving the antitumour activity of CAR-T-cells [133]

CAR is a kind of engineering, which can enable lymphocytes to identify and eliminate cells delivering homologous target ligands. It has antigen binding domain, hinge, transmembrane domain and intracellular signal domain modules. By changing each component, its function and anti-tumour effect can be adjusted. At present, various types of CARs are being developed and designed to improve the safety and effectiveness in cancer treatment [130]. Clinically, treatment with CAR-T-cells first requires T-cells, which can be obtained from the patient’s peripheral blood, or allogeneic CAR-T-cells obtained from a donor [131]. T-cells are stimulated and expanded in vitro and transduced with specific CAR genes through viral vectors, and then, the CAR-T-cells are infused back into the patient to perform the set tumour-killing effect in the patient's body. This type of therapy is also called CAR-T-cell therapy (Fig. 3).

In 2017, the FDA approved two types anti-CD19 CAR-T-cell products to treat both B-cell ALL and diffuse large B-cell lymphoma, and these products have transformed the field of anticancer immunotherapy [134]. However, there are still some limitations in CAR-T-cell therapy. Mechanisms hampering CAR-T-cell efficiency include limited T-cell persistence and therapy-related toxicity. Furthermore, severe toxicities, restricted trafficking to, infiltration into and activation within tumours, antigen escape and heterogeneity; manufacturing issues; physical properties; and the immunosuppressive capacities of solid tumours have prevented the success of CAR-T-cells in these entities [135, 136]. Additionally, it may not be possible to obtain a sufficient number of T-cells from the patient because the patient is usually not considered for CAR-T-cell therapy, usually due to a reduction in the number of original lymphocytes caused by previous cytotoxic treatment [134]. In application, almost all CAR-T-cell products are derived from CD4^+^ T-cells and CD8^+^ T-cells, both cell populations likely contribute to treatment effect [134]. Some plant extracts have beneficial effects on CD4^+^ T-cells and CD8^+^ T-cells. For example, Fuzheng Qingjie [137, 138], Fuzheng Fangai [139], Xiaoji [13], Cistanche deserticola [140], *Epimedium koreanum* Nakai [141], *Glycyrrhiza uralensis* [142, 143], *Aidi* [144], and *Scolopendra subspinipes* [145–147] can increase in CD4^+^ cells and the CD4/CD8 ratio, and produce FN-g, IL-2, IL-4, IL-6, and IL-7; Xiao Ai Ping [148], *Lycium barbarum* [149–151], Dangguibuxue tang [152], *Oldenlandia diffusa* [153–155], *Carthamus tinctorius* [156], lectin-55 [157], and *Tricosanthes kirilow* [158] have an effect on increasing in CD8^+^ cells, and tumour infiltration and increasing IFN-g and IL-10. Shenqi Fuzheng [159], *Lycium barbarum* [160], *Ganoderma lucidum* [161], Yunzhi-Danshen [162] can upregulate CD3^+^, CD4^+^, CD4 + /CD8 + and NK^+^ cells. Moreover, gastrodin was found to ameliorated the CD8^+^ T-cell-mediated immune response and significantly improved protection in tumour-challenged animals. This finding indicates that gastrodin is a potential adjuvant contributing to anticancer immunomodulation.

On the other hand, the tumour microenvironment is a complex pathological system composed of tumour cells, blood/lymphatic vessels, tumour stroma, and tumour-infiltrating myeloid precursors, providing a living environment for tumour cells and promoting tumour metastasis. In the tumour microenvironment, tumour-infiltrating myeloid precursors mainly include tumour-associated macrophages, tumour-associated dendritic cells, and myeloid-derived suppressor cells, which inhibit T-cells or other immune cells and play an important role in its antitumour activity. Therefore, improving the tumour microenvironment by targeting these cells is an effective way to assist CAR-T-cell therapy. Liu J et al. reviewed Chinese herbal medicine and its components that induce tumour cell apoptosis and directly inhibit tumour growth and invasion, providing new research ideas for cell therapy [163].

### TCR-T

Due to the limitations of CAR in the application, it only recognizes cell surface protein antigens, while TCR can distinguish intracellular proteins expressed as peptides on MHC class I molecules. Therefore, TCR-T therapy has superiority in the field of solid tumour treatment. The TCR can be produced in two ways. One method is to identify and clone T- cells from patients with antitumour reactions. Their TCRs are inserted into retroviruses or lentiviruses to infect target T-cells. Another method is to isolate TCRs from humanized mice that recognize tumour antigens. TCRs can be immunized with appropriate tumour antigens because they can express human MHC class I or II. After T-cells were isolated, the TCR gene was cloned into a recombinant vector for genetic engineering transformation of patients' autologous T-cells [164].

Although effective responses have been observed in TCR-T-cell therapy, adverse reactions have become a thorny issue in many trials. Most of the reasons are that TCR-T- cells, in addition to their killing effect on tumour cells, severely destroy normal cells with the same antigen [129]. Since TCR-T-cells have only emerged in recent years, there are almost no plant extracts currently used in TCR-T-cell research. Parvifoline AA is an ent-kaurane diterpenoid and can significantly stimulate the level of NKG2D ligands on hepatocellular carcinoma cells, evidently enhancing their recognition and lysis by NK cells [14]. Perhaps improving the efficacy of TCR-T-cells in the immunosuppressive microenvironment and determining that the expression is mainly (if not completely) limited to cancer cell targets may be a future research direction for plant extracts.

## Conclusion

Plant extracts are relatively easy to obtain and have significant activity in the treatment of many diseases. The above review shows that plant extracts have an effect on stem cell proliferation or directed differentiation and play an important role in solving the problem of insufficient endogenous stem cells and directed differentiation of stem cells; In immune cell therapy, the effect of plant extracts on stem cells are reflected in the beneficial effects on CD4^+^ T-cells and CD8^+^ T-cells and the improvement of the tumour microenvironment. Moreover, plant extracts, such as astragaloside [165], paeoniflorin [166], and licorice [167], have a good immunoregulatory and anti-inflammatory activities and may provide a better treatment plan for the cytokine storm caused by cell therapy [168]. At present, cell therapy is promising. However, to understand the long-term effects, more in-depth research on the dose and side effects of plant extract applications is still needed. Although plant extracts are recognized as excellent alternatives to synthetic interventions, clinical application is challenging due to the variability and complexity of the bioactive components present in the extracts, as well as the effects of solvents during extraction. Therefore, the effects of plant extracts on cell therapy need to be better and more deeply researched to supplement the current deficiencies in cell therapy.

## Supplementary Information

**Additional file 1: Table S1.** Plant Extracts for the Osteogenesis of MSCs.

## Data Availability

Not applicable.

## Abbreviations

MSCs: Mesenchymal stem cells
NSCs: Nerve stem cells
CAR-T: Chimeric antigen receptor T-cell immunotherapy
TCR-T: T-cell receptor modified T-cell immunotherapy
ASCs: Adipose-derived stem cells
iPSCs: Induced pluripotent stem cells
ESCs: Embryonic stem cells
HLA-DR: Human leukocyte antigen-antigen D related
BM-hMSCs: Bone marrow-derived human MSCs
ERK: Extracellular signal-regulated kinase
Runx2: Runt-related transcription factor 2
HGPS: Hutchinson–Gilford progeria syndrome
VEGF: Vascular endothelial growth factor
VEGFR: Vascular endothelium growth factor receptor
TCM: Traditional Chinese medicine
ALP: Alkaline phosphatase
PPARγ: Peroxisome proliferator activated receptor γ
BSNXD: BuShenNingXin decoction
WS-MSCs: Wharton's jelly-derived MSCs
C/EBP-α: CCAAT enhancer-binding protein-α
Smo: Smoothened
OGD: Oxygen and glucose deprivation
bFGF: Basic fibroblast growth factor
NSPC: Neural stem/progenitor cells
GFAP: Glial fibrillary acidic protein
*n*-BP: Butylidenephthalide
ET-1: Endothelin 1
BNP: Brain natriuretic peptide
UVB: Ultraviolet-B
EpSCs: Epidermal stem cells
ACT: Adoptive cell therapy
