# Supplementary material for: The potential of plant extracts in cell therapy
Source: Stem Cell Res Ther. 2022 Sep 14;13:472. doi: 10.1186/s13287-022-03152-z (PMC9476258; doi:10.1186/s13287-022-03152-z)
Supplement: Supplementary file 1 — Additional file 1: Table S1. Plant Extracts for the Osteogenesis of MSCs. [file 13287_2022_3152_MOESM1_ESM.docx]

**Table S1** Plant Extracts for the Osteogenesis of MSCs

| **NO.** | **Plant extracts/ phytohemicals** | **Cell lines** | **Mechanism and Pathway** | **Ref** |
| --- | --- | --- | --- | --- |
| 1 | Amentoflavone | hMSCs | promote osteogenesis by stimulating ALP activity and mineralization in hMSCs, with the involvement of JNK and p38 MAPK pathways | [18] |
| 2 | *Foeniculum vulgare* | BM-hMSCs | increase of BM-hMSC proliferation and differentiation into osteoblasts | [16] |
| 3 | *Ferula gummosa* | BM-hMSCs | proliferate and differentiate BM-hMSCs into osteocytes, demonstrating increased alkaline phosphatase activity | [17] |
| 4 | Dipsacus asper (hedraganin-3-O-(2-O-acetyl)-α-L-arabinopyranoside) | BM-hMSCs | enhance osteoblastic differentiation due to induced alkaline phophatase activity and expression of bone-specific proteins (bone sialoprotein and osteocalcin) | [26] |
| 5 | *Fructus Ligustri Lucidi* | BM-hMSCs | increase the expression of osteogenic stimulating genes, β-catenin, BMP2, cyclin D1, MT1-MMP, os-teoprotegerin and TBX3 | [27] |
| 6 | Poncirin | C3H10T1/2 MSCs and primary bone marrow MSCs | upregulate the expression of the osteoblastic marker genes in BM-MSCs, and contribute to the osteoblast mineralization at the late stages of differentiation; inhibited the expression of adipogenic marker genes, such as PPAR-γ and C/EBP- β | [28] |
| 7 | *Panax notoginseng* saponins | MSCs | increased ALP activity, Alizarin red S staining and mRNA level of ALP, Cbfa 1, OC, and BSP, whereas decreased the mRNA level and protein expression of PPARγ2 during osteogenic induction | [29] |
|  |  | MSCs | enhanced the mRNA expression of OPG, β-catenin, and cyclin D1 while decreased the mRNA expression of RANKL and PPARγ2 | [30] |
| 8 | Naringin | BM-hMSCs | increased mRNA levels of osteogenic genes and Notch1 expression, while decreasing PPARγ2 mRNA levels | [31] |
|  |  | BM-MSCs | enhance the proliferation and osteogenic differentiation of human BM-MSCs | [32] |
|  |  | human ASCs | activation of Wnt signaling | [44] |
| 9 | Harmine | C3H10T1/2 cells | promoted ALP activity in MC3T3-E1 cells without affecting their proliferation; increased the mRNA expressions of the osteoblast marker genes ALP and osteocalcin;enhanced the mRNA expressions of Runx2 and Osterix, which are key transcription factors in osteoblast differentiation; inducing the expressions of BMPs and activating BMP and Runx2 pathways | [33] |
| 10 | Epigallocatechin-3- gallate | BM-hMSCs | promote cell growth; enhance osteogenesis in the presence of osteoinductive agents through the upregulation of BMP2 expression | [34] |
| 11 | Gastrodin | BM-hMSCs | promoted the proliferation,improved ALP, OCN, COL I, OPN; reduced ROS | [19] |
| 12 | ZD-I | hMSC-TERT cell | stimulatory effects on the proliferation and inhibitory effects on mineralization of hMSCs through down-regulation of several osteogenic markers such as osteocalcin, BMP-2, and osteopontin in the late stages | [24] |
| 13 | BuShenNingXin Decoction | mice MSCs | increased ALP activity as well as collagen type I, osteocalcin, Runx2, and osterix mRNA expression; decreased adipocyte numbers and PPARγ mRNA expression, whereas in Tregs; enhanced ALP activity | [37] |
| 14 | Flavonoids of epimedii | BM-hMSCs | increased rates of osteogenic activity through the BMP or Wnt-signaling pathway | [38] |
| 15 | *Angelica sinensis* polysaccharide | rat BMSCs | promote the osteogenic differentiation of rat BM-MSCs under high glucose culture and induce bone regeneration in rats with type 2 diabetes; activation of the Wnt/β-catenin signaling pathway. | [39] |
| 16 | *Ginkgo biloba* (Ginkgolide B) | BM-hMSCs | promote osteogenesis in hBMSCs through up-regulation of Runx2 and BMP4 expression in BMP signalling, as well as β-catenin and Cyclin D1 in Wnt/β-catenin signalling; Ginkgolide B: Wnt/β-catenin-dependent | [40][45] |
| 17 | Berberine | BM-MSCs | stimulate osteogenic differentiation of MSCs by enhancing Runx2 expression and by activating canonical Wnt/β-catenin signaling pathway | [41] |
| 18 | Salvianolic acid B | BM-MSCs | influence the ERK signalling pathway; affected Wnt pathway through the up-regulation of β-catenin mRNA expression and down-regulation of Dickkopf-1 (DKK-1); enhanced Runx2 and decreased PPARγ mRNA expression, promoting the lineage commitment of MSCs to osteogenesis | [42] |
|  |  | MSCs | stimulated MSC differentiation to osteoblast and increased osteoblast activities, decreased GC associated adipogenic differentiation by down-regulation of PPARγ mRNA expression, increased Runx2 mRNA expression without osteoblast inducement, decreased Dickkopf-1 and increased β-catenin mRNA expression with or without adipocyte inducement in MSC | [43] |
| 19 | Silibinin | BM-hMSCs | inducing the expressions of BMPs and activating BMP and Runx2 pathways | [46] |
| 20 | Genistein | BM-hMSCs | the differentiation mediated by ERα-dependent BMP activation leading to increasing expression of Runx2 and small mothers against decapentaplegic homologs 5 (SMAD 5) | [47] |
| 21 | Icariin | rat BM-MSCs | increased ERK, p38 kinase and JNK | [49] |
|  |  | MSCs | activation of the PI3K-AKT-eNOS-NO-cGMP-PKG pathway in treated cells | [50] |
| 22 | Quercetin | BM-MSCs | activation of ERK and p38 signalling pathways | [51] |
|  |  | hMSCs | decrease in replicative senescence, oxidative stress, inflammation and apoptosis in WS-MSCs; enhancement of osteogenic and chondrogenic differentiation in WS-MSCs; attenuation of cellular senescence in HGPS MSCs and in both physiological-aging MSCs | [52] |
|  |  | ASCs | increase osteogenic differentiation of mouse ASCs, enhancing the expression of Osx, Runx2, BMP- 2, Col1, OPN and OCN genes | [36] |
| 123 | Fucoidan | BM-hMSCs | induces osteoblast differentiation through BMP2-Smad 1/5/8 signaling by activating ERK and JNK | [53] |
|  |  | murine BM-MSCs | promote osteogenic differentiation via controlling the sequential markers at the early and late stages of osteogenic differentiation in D1 cells | [54] |
|  |  | human ASCs | enhanced the expression of osteogenesis-specific marker genes, including ALP, osteopontin, type I collagen, Runt-related transcription factor 2, and osteocalcin | [35] |
| 24 | Resveratrol | MSCs and pre-osteoblastic cells | Runx2 acetylation/deacetylation | [55] |
|  |  | BM-hMSCs | stimulates BM-hMSCs proliferation and osteoblastic differentiation through an ER-dependent mechanism and coupling to ERK1/2 activation | [20] |
|  |  | hMSCs | promotes cell self-renewal by inhibiting cellular senescence,and inhibits cell self-renewal by increasing senescence rate, cell doubling time and S-phase cell cycle arrest | [56] |
| 25 | Du-Huo-Ji-Sheng-Tang (Ligusticum chuanxiong) | hMSCs | Ligusticum chuanxiong increased osteogenic activity in hMSCs and up-regulated BMP-2 and RUNX2 gene expression via the activation of SMAD 1/5/8 and ERK signaling | [48] |
